# SMYD2 targets RIPK1 and restricts TNF-induced apoptosis and necroptosis to support colon tumor growth

**DOI:** 10.1038/s41419-021-04483-0

**Published:** 2022-01-12

**Authors:** Yu-qiang Yu, Veronika Thonn, Jay V. Patankar, Oana-Maria Thoma, Maximilian Waldner, Marta Zielinska, Li-li Bao, Miguel Gonzalez-Acera, Stefan Wallmüller, Felix B. Engel, Michael Stürzl, Markus F. Neurath, Eva Liebing, Christoph Becker

**Affiliations:** 1 Department of Medicine 1, Friedrich-Alexander-Universität Erlangen-Nürnberg (FAU), Erlangen, Germany; 2 Deutsches Zentrum Immuntherapie (DZI), 91054 Erlangen, Germany; 3 Department of Biochemistry, Faculty of Medicine, Medical University of Lodz, Łódź, Poland; 4 Experimental Renal and Cardiovascular Research, Department of Nephropathology, Institute of Pathology, Friedrich-Alexander-Universität Erlangen-Nürnberg (FAU), Erlangen, Germany; 5 Comprehensive Cancer Center Erlangen-EMN (CCC ER-EMN), 91054 Erlangen, Germany; 6 Division of Molecular and Experimental Surgery, Department of Surgery, Friedrich-Alexander-Universität Erlangen-Nürnberg (FAU), 91054 Erlangen, Germany

**Keywords:** Colon cancer, Apoptosis

## Abstract

SMYD2 is a histone methyltransferase, which methylates both histone H3K4 as well as a number of non-histone proteins. Dysregulation of SMYD2 has been associated with several diseases including cancer. In the present study, we investigated whether and how SMYD2 might contribute to colorectal cancer. Increased expression levels of SMYD2 were detected in human and murine colon tumor tissues compared to tumor-free tissues. SMYD2 deficiency in colonic tumor cells strongly decreased tumor growth in two independent experimental cancer models. On a molecular level, SMYD2 deficiency sensitized colonic tumor cells to TNF-induced apoptosis and necroptosis without affecting cell proliferation. Moreover, we found that SMYD2 targeted RIPK1 and inhibited the phosphorylation of RIPK1. Finally, in a translational approach, pharmacological inhibition of SMYD2 attenuated colonic tumor growth. Collectively, our data show that SMYD2 is crucial for colon tumor growth and inhibits TNF-induced apoptosis and necroptosis.

## Introduction

Colorectal cancer (CRC) is one of the leading causes of cancer-deaths worldwide and is expected to increase by 60% by the year 2030 [1, 2]. Multiple approaches including surgical removal and chemotherapy have been applied in CRC treatment, but the prognosis of patients with advanced CRC remains poor [3]. In order to improve the clinical management of CRC, it is crucial to identify the molecular mechanisms involved in CRC development and novel targets of anti-tumor therapy.

The SET and MYND domain-containing protein 2 (SMYD2) catalyzes the lysine methylation of histone and non-histone proteins to regulate multiple biological processes in tumor progression [4–6]. SMYD2 has been shown to regulate two opposing processes, cell proliferation and apoptosis [7–9]. The proliferation of tumor cells is crucial for the promotion of the tumor development [10]. Recently, SMYD2 has been implicated in regulating cell proliferation via targeting various molecules [9, 11, 12]. For example, SMYD2 physically interacts with proteins in the TGFβ pathway, including SMAD1–4, SMAD7, and SMURF2 [13]. Moreover, EZH2, a transcriptional repressor of several anti-proliferative genes, has been shown to undergo methylation by SMYD2, leading to EZH2 stability and therefore enhancing cell proliferation in breast cancer [6, 14]. Apoptosis is a form of programmed cell death and its inhibition plays an important role in CRC tumor development [15, 16]. Progressive inhibition of apoptosis is associated with the transformation of colorectal epithelial to carcinoma cells and decreased apoptosis has been observed in CRC tissues [17, 18]. Apoptosis can be triggered via an extrinsic death receptor pathway or by an intrinsic mitochondrial pathway [19]. The extrinsic pathway is triggered upon binding of death receptor ligands to their specific death receptors. TNF, a well-known death ligand, binds to TNFR1 to recruit downstream molecules such as RIPK1, leading to apoptotic pathway activation [20]. On the other hand, the intrinsic pathway is induced by intrinsic signaling such as DNA damage or endoplasmic reticulum stress. Accordingly, SMYD2 was shown to be required to limit the p53-mediated intrinsic apoptotic pathway in cardiac cells [7]. However, it is currently unknown whether SMYD2 may also regulate apoptosis via an extrinsic pathway.

Here we discovered that SMYD2 deficiency in human or murine colon tumor cells compromised tumor growth in orthotopic and non-orthotopic CRC models. Using MC-38 and HT-29 cells, we demonstrated that SMYD2 is dispensable for cell proliferation but crucial for inhibiting TNF-induced apoptosis and necroptosis. Our analysis further revealed that SMYD2 deficiency increased RIPK1 phosphorylation. Finally, we proved that the pharmacological inhibition of SMYD2 using two different SMYD2 inhibitors, AZ505 and BAY-598, attenuates colon tumor growth. Taken together, we identified SMYD2 as an important molecule for colon tumor growth via regulating TNF-induced apoptosis and necroptosis.

## Results

### SMYD2 is highly expressed in CRC and its deficiency reduces colon tumor growth in vivo

**Fig. 1:**
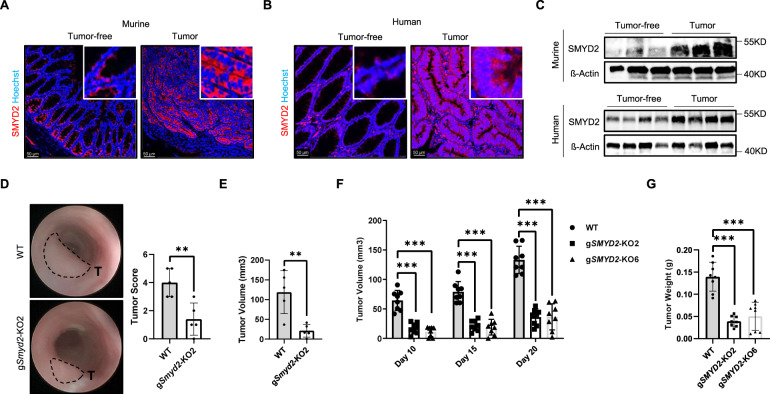
SMYD2 is highly expressed in CRC and SMYD2 deficiency reduces colon tumor growth in vivo. Immunofluorescence staining of murine Apc^Min/+^ (**A**) or human (**B**) colon tumor and tumor-free tissues with anti-SMYD2 antibody. Hoechst was used to stain the nuclei. **C** Immunoblot analysis of SMYD2 in lysates of human or murine colon tumor and tumor-free tissues. β-Actin served as a loading control. **D**, **E** WT and *Smyd2* CRISPR/Cas9 knockout MC-38 cells were implanted into mice via submucosal injection. **D** Representative images of the colonic tumors and miniendoscopy-assisted tumor scoring. **E** Measurements of colonic tumor volume upon euthanasia. **F** Tumor volume from mice in which CRISPR/Cas9-directed *SMYD2* knockout or WT HT-29 cells were injected subcutaneously and **G** tumors weight at day 20 from mice shown in (**F**) above.

In order to investigate a potential contribution of SMYD2 in colorectal cancer (CRC) development, we initially detected the expression pattern of SMYD2 in colon tumors in murine samples (Fig. 1A). Immunofluorescence staining revealed SMYD2 staining in intestinal epithelial cells, in particular within the crypt region. Interestingly, SMYD2 staining was strongly increased in tumor tissues compared to tumor-free adjacent tissues. A similar increase in SMYD2 levels was observed in tissues derived from human CRC patients (Fig. 1B). Western blot analysis for SMYD2 further confirmed these findings (Fig. 1C). These data show that SMYD2 is highly expressed in human and murine colon tumors.

To further elucidate a functional contribution of SMYD2 during CRC tumor development, SMYD2 deficient MC-38 cells and HT-29 cells were generated using the CRISPR/Cas9 technology (Fig. S1A). The role of SMYD2 in colorectal carcinogenesis was investigated by subcutaneously injecting the MC-38 *Smyd2* deficient and WT (wild type) cells into C57BL/6J mice. Tumor growth was monitored at different time points and tumor weight was determined at the experimental end point. Interestingly, *Smyd2* deficient tumors showed a strong reduction in the volume and weight compared with WT tumors (Figs. S1B and S1C). To exclude the possibility that the effects may depend on the nonphysiologic site of injection, we next implanted MC-38 WT and *Smyd2* deficient cells directly into the colonic submucosa. Tumor size was monitored and scored via colonoscopy (Fig. 1D). When implanted into the submucosa, the *Smyd2* deficient MC-38 cells resulted in tumors that had a significantly reduced tumor size and lower tumor scores (Fig. 1D, E), which was similar to the results obtained from the subcutaneous injection experiment. In summary, our results, from orthotopic and non-orthotopic transplantation of MC-38 WT and *Smyd2* deficient cells, suggesting an important role of SMYD2 in murine colon tumor growth.

Given the above findings, we studied whether the role of SMYD2 in regulating colon tumor growth is conserved in humans. With this aim, *SMYD2*-proficient and -deficient HT-29 cells were implanted into *Rag1*^−/−^ mice via subcutaneous injection, and tumor development was monitored over time. Similar to results obtained from the experiments performed with murine tumor cells, *SMYD2* deficient HT-29 tumors showed a significant decrease in tumor size and tumor weight (Fig. 1F, G). Taken together, these data demonstrated that *Smyd2* deficiency reduces murine colon tumor growth in vivo and that this process is conserved in human colorectal cancer cells.

### SMYD2 is dispensable for CRC proliferation

**Fig. 2:**
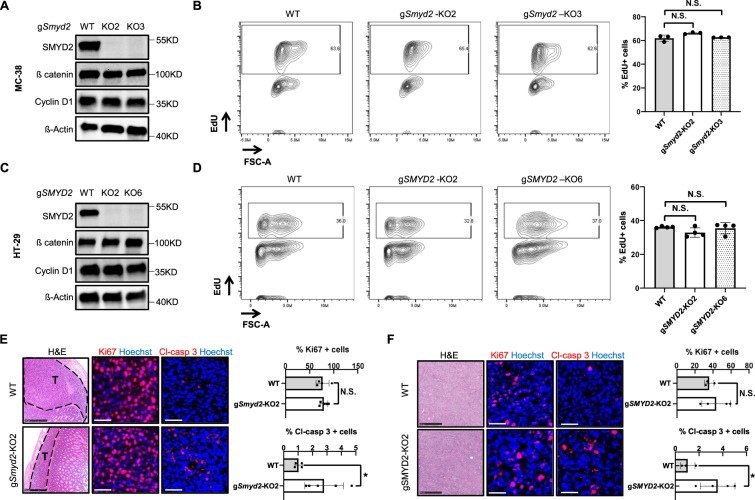
Proliferation is not impeded in SMYD2 deficient CRC cells. Immunoblot analysis of SMYD2, Cyclin D1, and ß catenin in lysates of WT and two clones of CRISPR/Cas9-directed SMYD2 knockout MC-38 cells (**A**) or HT-29 cells (**C**). β-Actin served as a loading control. Flow cytometry assay of EdU in WT and SMYD2 CRISPR/Cas9 knockout MC-38 cells (**B**) or HT-29 cells (**D**). Graph shows quantification of EdU-positive cells. Experiments were performed three times and representative data are shown. Data are presented as mean+SD and student’s t-test was used for statistical calculation. N.S., not significant. Representative images of H&E, Ki67 (red), and cleaved-caspase 3 (red) stained MC-38 tumor tissues (**E**) or HT-29 tumor tissues (**F**). Scale bar, 50 µm. Histograms in **E** and **F** show quantification of Ki67 or cleaved-caspase 3 positive cells in tumor tissues. *n* (MC-38 WT) = 4, *n* (MC-38 g*Smyd2*-KO2) = 5, *n* (HT-29 WT) = 3, *n* (HT-29 g*SMYD2*-KO2) = 4.

Recently, SMYD2 has been shown to regulate proliferation in multiple cancer cells lines [21]. We, therefore, assessed the effects of SMYD2 deficiency on colon tumor cell proliferation. Interestingly, loss of *Smyd2* in MC-38 cells had no effect on the protein levels of ß catenin and Cyclin D1, two important proliferation markers (Fig. 2A). We next analyzed DNA synthesis in cultured MC-38 cells via 5-Ethynyl-2´-deoxyuridine (EdU) assay. In line with the findings above, we detected similar numbers of EdU-positive cells in the *Smyd2* deficient group compared with the WT MC-38 group (Fig. 2B), collectively suggesting that SMYD2 is dispensable for regulating cell proliferation in MC-38 cells. In order to investigate whether this observation is a MC-38 cell-specific effect of SMYD2, we further analyzed *SMYD2* knockout HT-29 cells. Similar to the results obtained from murine cells, *SMYD2* deficiency in human HT-29 cells had no effects on protein levels of ß catenin and Cyclin D1 (Fig. 2C). Comparable levels of EdU-positive cells were observed in WT and *SMYD2* deficient HT-29 cells. In summary, our data show that SMYD2 is dispensable for CRC cell proliferation in vitro.

We then studied whether SMYD2 regulates CRC cell proliferation in vivo via detecting Ki67-positive cells in tumor tissues from the orthotopic and xenotopic transplant models. Comparable numbers of Ki67-positive cells were detected in WT and *Smyd2* deficient MC-38 tumor implants (Fig. 2E) and also from the WT and *SMYD2* deficient HT-29 tumor implants (Fig. 2F), suggesting that SMYD2 is dispensable for regulating cell proliferation in vivo. To our surprise, we found a significant elevation of cleaved-caspase 3 positive cells in the tumor implants of MC-38 and HT-29 SMYD2 deficient cells compared with MC-38 and HT-29 WT cell implants (Fig. 2E, F). Taken together, these data indicate that SMYD2 is dispensable for CRC cell proliferation but restricts apoptosis in vivo.

### SMYD2 deficiency sensitizes CRC cells to TNF-induced apoptosis and necroptosis

**Fig. 3:**
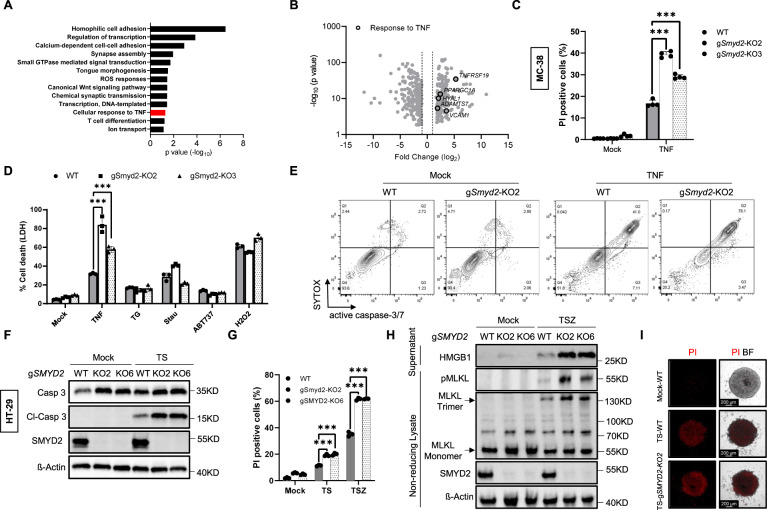
SMYD2 deficiency sensitizes CRC cells to TNF-induced apoptosis and necroptosis. **A** Gene ontology analysis for biological processes among the significantly and commonly regulated genes between the two clones of *SMYD2* deficient HT-29 implanted tumors compared against WT HT-29 implanted tumors. Cellular response to TNF (highlighted red) among the significantly upregulated biological processes. **B** Volcano plot showing the expression of significantly downregulated and upregulated genes in *SMYD2* deficient tumors compared to WT tumors. Black circles highlight genes related to TNF response. **C** Flow cytometric quantification of Propidium iodide (PI) stained WT and two different clones of *Smyd2* deficient MC-38 cells stimulated with 0.1 ng/ml TNF or vehicle (mock). **D** LDH release from WT and *Smyd2* CRISPR/Cas9 knockout MC-38 cells stimulated with vehicle (Mock), 0.1 ng/ml TNF, 25 µM thapsigargin (TG), 5 µM staurosporine (Stau), 50 µM ABT737, or 0.5 mM H_2_O_2_ for 24 h. **E** Flow cytometric detection of cleaved caspase-3/7 activity and SYTOX from the indicated genotypes of MC-38 cells stimulated with 0.1 ng/ml TNF. **F** Immunoblot analysis of SMYD2, caspase 3, and Cl-caspase 3 in HT-29 cells treated with or without 40 ng/ml TNF + 4 nM LCL161 (TS). β-Actin served as a loading control. **G** Flow cytometric quantification of PI stained HT-29 cells stimulated with TS or 10 ng/ml TNF + 1 nM LCL161 + 20 µM Z-VAD (TSZ). **H** Immunoblot analysis of HMGB1 from the supernatants and pMLKL/MLKL from non-reducing cell lysates. WT and SMYD2 CRISPR/Cas9 knockout HT-29 cells were stimulated with or without TSZ. β-Actin served as a loading control. **I** Representative images of PI stained HT-29 cells that were cultured in 3D. WT and *SMYD2* CRISPR/Cas9 knockout HT-29 cells were stimulated with or without TS. DMSO was used as control. Experiments were performed three times and representative data are shown. Data are presented as mean +SD and student’s t-test was used for statistical calculation. **P < 0.01 and ***P < 0.001. N.S. not significant.

To further narrow down the mechanism of cell death induction upon SMYD2 deficiency, RNA-seq analysis was performed on implanted tumor tissues from two clones of *SMYD2* deficient and WT HT-29 cells. We found 163 upregulated and 215 down-regulated genes that were common between the two knockout clones compared with WT (Fig. S2A; full gene list is given in Supplementary Table 1). A significant enrichment was found in the gene ontology for the biological process of cellular response to TNF (Fig. 3A). Furthermore, several TNF-related genes including *TNFRSF19, VCAM1, and ADAMTS17* were highly upregulated in *SMYD2* deficient cells (Fig. 3B). Based on these data together with the elevated cleaved-caspase 3 positivity in SMYD2 deficient tumors, we hypothesized that SMYD2 deficiency sensitizes colon tumor cells to TNF-induced apoptotic cell death. To evaluate this hypothesis, WT and two different *Smyd2* deficient clones of MC-38 cells were stimulated with TNF in vitro and then stained with cell-impermeable propidium iodide (PI) which stains dead cells. Interestingly, the percentage of PI-positive cells was significantly higher in both clones of *Smyd2* deficient MC-38 cells compared to WT cells following TNF stimulation (Figs. 3C and S2B), suggesting that SMYD2 plays a role downstream of TNF in regulating cell death. We next confirmed this finding via detecting the release of LDH, a prototypical proxy for cell membrane rupture used to detect cell death. MC-38 cells were stimulated with various inducers of cell death (Fig. 3D). As expected, challenging WT MC-38 cells with TNF, thapsigargin, staurosporine, ABT737, and H_2_O_2_ induced significant cell death. Interestingly, *Smyd2* deficiency specifically and significantly amplified the TNF-induced cell death but not the cell death induced by other stimuli. In order to confirm that this is apoptotic cell death, we performed a flow cytometry analysis by detecting activated caspase-3/7 and SYTOX, where double positive cells were categorized as apoptotic cells (Figs. 3E and S2C). After TNF stimulation, we observed *Smyd2* deficient MC-38 cells showed a highly significant elevation in the number of apoptotic cells compared with WT MC-38 cells (Figs. 3E and S2C). Moreover, the presence of Z-VAD, a pan-caspase inhibitor, reduced TNF-induced apoptosis in WT cells and abolished the increased cell death observed in *Smyd2* deficient MC-38 cells (Fig. S2D). These results suggested that SMYD2 regulates TNF-induced apoptosis via a caspase-dependent pathway in MC-38 cells.

In order to further investigate the effect of SMYD2 in human cells, we also induced apoptosis in *SMYD2* proficient and deficient HT-29 cells. The LCL161, a classical second mitochondria-derived activator of caspase (SMAC) mimetic, was used together with TNF to induce RIPK1-dependent apoptosis in HT-29 cells. As expected, TNF plus LCL161 (TS) treatment induced significant caspase 3 activation in HT-29 cells (Fig. 3F). Interestingly, *SMYD2* deficiency increased levels of cleaved-caspase 3 upon TS stimulation in both the clones of *SMYD2* deficient cells to levels that were higher than those seen in *SMYD2* sufficient HT-29 cells. This finding was further confirmed via flow cytometry for PI-positive cells in TS treated HT-29 cells (Fig. 3G). These data indicate that SMYD2 modulates the TNF-RIPK1 signaling arm in HT-29 cells.

A key feature of tumor cells is the ability to evade programs of cell death and one of the strategies employed is the evasion of necroptosis via the activation of initiator caspase modulators such as c-FLIP. We recently showed that forced necroptosis induction in CRC cells downstream of TNF can be a successful strategy to counter such cell death evasion [22]. To address whether SMYD2 is a modulator of the necroptosis arm downstream of TNF, we stimulated HT-29 cells with TS plus Z-VAD (TSZ) (Fig. 3G). As expected, TSZ treatment induced cell death in HT-29 WT cell cultures. Interestingly, we observed a significantly higher number of PI-positive cells in *SMYD2* deficient cells, suggesting that SMYD2 inhibits necroptosis. In line with the elevated necroptosis in cells lacking *SMYD2*, we also observed an increase in the levels of HMGB1 release and MLKL trimerization (Fig. 3H), two biomarkers of necrotic cell death [23, 24]. Furthermore, the presence of necrostatin-1 (Nec-1), a specific inhibitor of RIPK1 kinase activity, prevented TSZ-induced cell death both in WT cells as well as in *SMYD2* deficient cells (Fig. S2E). Similarly, RIPK3 inhibition (GSK-872) or MLKL inhibition (NSA: Necrosulfonamide) were both efficient in blocking TSZ-induced cell death in *SMYD2*-deficient HT29 cells (Fig. S2E). Interestingly, more pMLKL positive cells were detected in *SMYD2* deficient HT-29 tumors in vivo (Fig. S2F). Taken together, our data indicated that SMYD2 regulates necroptosis induction in HT-29 cells. Of note, TSZ treatment couldn’t induce cell death in MC-38 cells (Fig. S2G, upper panel). The necroptosis insensitivity of MC-38 cells is likely due to the deficiency of RIPK3 (Fig. S2G, lower panel) [25], a key molecule for necroptosis induction.

The studies described above were performed on 2D-cultured cells and 2D cell culture lacks many aspects of tumors, such as hypoxia and altered cell-cell contacts [26–28]. Therefore we stimulated 3D cultures of HT-29 cells with TS to mimic the in vivo tumor response upon an apoptotic challenge (Figs. 3I and S2H). Similar to the results obtained from the experiments performed in 2D culture of HT-29 cells, *SMYD2* deficiency significantly increased cell death induced by TS stimulation. In summary, these data demonstrated that SMYD2 regulates both TNF-induced apoptosis and necroptosis.

### SMYD2 regulates RIPK1 recruitment and phosphorylation

The binding of TNF to TNFR1 leads to the recruitment of RIPK1 and a number of other molecules [29]. TNF stimulation induces the expression of pro-survival genes, such as those encoding FLIP or cIAP1/2, which suppress the activation of cell death pathways [29]. FLIP or cIAP1/2 deficiency switches cells from TNF-induced pro-survival pathways to TNF-induced cell death pathways. In the presence of caspase 8, a cytosolic complex named complex IIa can be further formed to induce RIPK1-dependent apoptosis. When caspase-8 activation is blocked, the signaling can be switched to form another complex, named complex IIb, to induce RIPK1-dependent necroptosis [29].

**Fig. 4:**
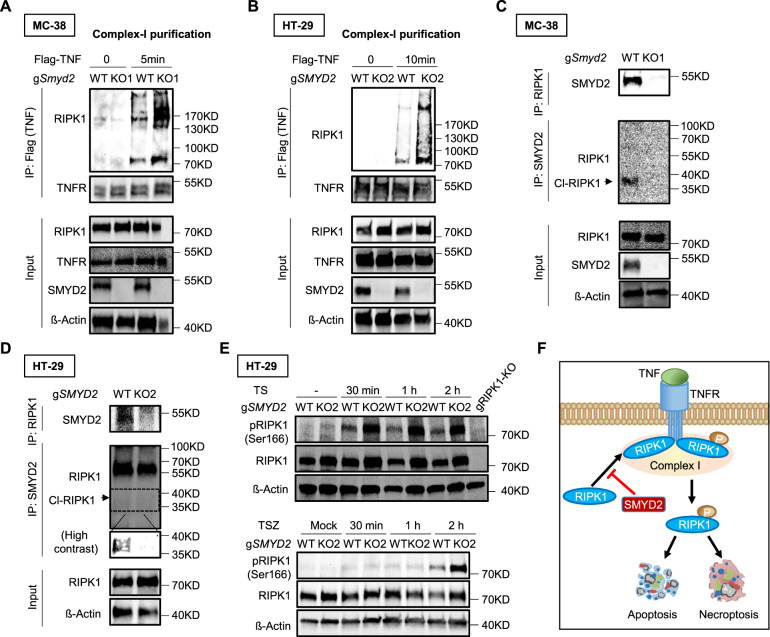
SMYD2 regulates RIPK1 recruitment and phosphorylation. Flag TNF-induced complex-I immunoprecipitation. MC-38 cells (**A**) or HT-29 cells (**B**) were stimulated with Flag-TNF (1 µg/mL) for the indicated time points, followed by Flag immunoprecipitation and immunoblot analysis. HT-29 cells were pre-treated with 1 nM LCL161 for 1 h before Flag-TNF stimulation cell lysates of MC-38 cells (**C**) or HT-29 cells (**D**) stimulated with or without TNF were immunoprecipitated with antibody against RIPK1 or antibody against SMYD2, followed by immunoblot analysis. HT-29 cells were pre-treated with 1 nM LCL161 for 1 h before TNF stimulation. **E** Immunoblot of phosphorylated or total RIPK1 in lysates of WT and *SMYD2* deficient HT-29 cells stimulated with TS (Upper panel) or TSZ (Lower panel) at different time points. β-Actin served as a loading control. **F** A schematic diagram depicting SMYD2 mediated cell death pathways in CRC cells.

Knowing that SMYD2 regulates TNF-induced apoptosis and necroptosis, we hypothesized that SMYD2 may target the RIPK1 recruitment. To test this hypothesis, we stimulated MC-38 cells with Flag-TNF and pulled-down complex I via Flag immunoprecipitation (Fig. 4A). By doing so, we detected enhanced levels of RIPK1, a key component in the complex I, in the immunoprecipitate of WT cells after TNF treatment (Fig. 4A). Interestingly, in *Smyd2* deficient cells, we discovered increased co-precipitation of RIPK1 upon TNF stimulation, suggesting that *Smyd2* deficiency promotes RIPK1 recruitment to a TNF/TNF receptor 1 complex. In order to further investigate whether this finding was restricted to MC-38 cells, we also stimulated HT-29 cells with Flag-TNF and purified complex I (Fig. 4B). Similar to the results obtained from experiments performed with murine MC-38 cells, *SMYD2* deficient HT-29 cells showed a significant increase of RIPK1 co-precipitation. Collectively, these data suggested that SMYD2 inhibits RIPK1 recruitment after TNF stimulation.

We then tested whether SMYD2 can interact with RIPK1. Cell lysates of MC-38 cells were immunoprecipitated with antibody against RIPK1 and then immunoblotted for SMYD2, or vice versa. *Smyd2* deficient cells were used as a negative control. Our results demonstrated an interaction between RIPK1 and SMYD2, as SMYD2 co-immunoprecipitated with RIPK1 (Fig. 4C). Interestingly, SMYD2 specifically interacted with cleaved-RIPK1, not full-length RIPK1, suggesting that SMYD2 regulates the apoptotic pathway via targeting cleaved-RIPK1. We further confirmed this finding in HT-29 cells (Fig. 4D). Since a crucial role of RIPK1 phosphorylation has been demonstrated in its activation [30], we hypothesized that SMYD2 may impact the degree of RIPK1 phosphorylation. Accordingly, we analyzed RIPK1 phosphorylation in HT-29 cells stimulated with TS for activating the apoptotic pathway, or with TSZ for activating the necroptotic pathway (Fig. 4E). As expected, both TS and TSZ stimulation-induced some phosphorylation of RIPK1 in WT cells. However, *SMYD2* deficient cells showed increased phosphorylation of RIPK1, suggesting that SMYD2 functions by inhibiting RIPK1 phosphorylation (Fig. 4E). Taken together, our results delineate a signaling pathway in which SMYD2 decreases RIPK1 phosphorylation, which in turn inhibits TNF-induced apoptosis and necroptosis (Fig. 4F).

### Pharmacological inhibition of SMYD2 attenuates colon tumor growth

So far, our data revealed a significant impact of SMYD2 in regulating CRC cell death to such an extent that the genetic loss of SMYD2 blocked tumor growth. In order to test whether this function of SMYD2 is therapeutically exploitable against CRC, we inhibited SMYD2 activity using AZ505, a specific SMYD2 inhibitor [8, 31]. The presence of AZ505 significantly increased TNF-induced cell death and RIPK1-SMYD2 interaction (Fig. S3).

**Fig. 5:**
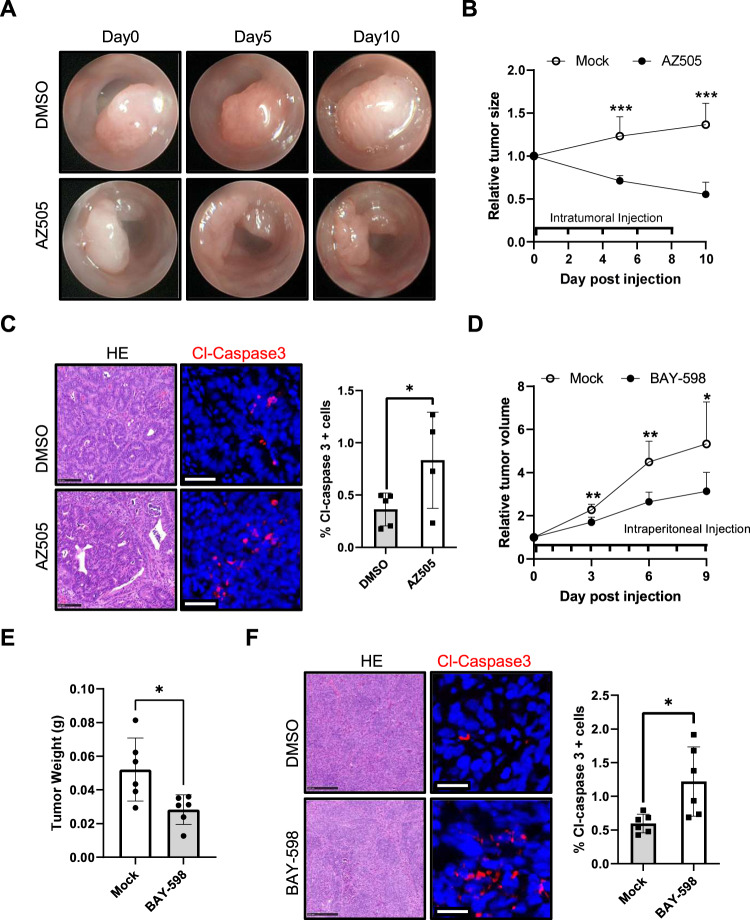
Inhibition of SMYD2 with AZ505 or BAY-598 suppressed colon tumor growth. **A**−**C** Apc^Min/+^ tumors were intratumorally injected with AZ505 or DMSO every two days. **A**, **B** Tumor size scored using colonoscopy at indicated time points (*n* = 5/group). **C** Representative images of H&E and cleaved-caspase3 (red) stained Apc^Min/+^ tumor sections (scale bar, 25 µm). The graph shows quantification of cleaved-caspase3 positive cells in tumor tissues. **D**, **E** HT-29 cells were injected subcutaneously into *Rag1*^−/−^ mice. Once tumor size was measureable, mice were intraperitoneal injected with BAY-598 or DMSO daily (*n* = 6/group). **D** Relative tumor volume monitored using calipers at three-day intervals. Tumor volume was normalized to the volume at the beginning of treatment. **E** Tumor weight at day 10 post euthanasia. **F** Representative images of H&E and cleaved-caspase3 (red) stained tumor sections (scale bar, 25 µm). The graph shows quantification of cleaved-caspase3 positive cells in tumor tissues.

Interestingly, local injection of AZ505 in Apc^Min/+^ mice via intratumoral injection led to a significant reduction in tumor size (Fig. 5A, B). What is more, elevated levels of cleaved-caspase3 positive cells were detected in AZ505-treated tumors compared with control tumors (Fig. 5C). Taken together, our data indicated that SMYD2 inhibition via AZ505 administration suppresses colon tumor growth.

To exclude the possibility of off-target effects of AZ505 or murine cell specific effects, we used BAY-598, an alternative SMYD2 inhibitor that has a different binding mode compared to AZ505 [32, 33], to further investigate the impact of SMYD2 inhibition in human colon tumor growth. HT-29 cells were injected subcutaneously into *Rag1*^−/−^ mice, and tumor development was monitored over time. BAY-598 treatment significantly inhibited tumor growth (Fig. 5D), reduced tumor weight (Fig. 5E), and increased levels of cleaved-caspase3 positive tumor cells (Fig. 5F). In summary, our results demonstrate that pharmacological inhibition of SMYD2 suppresses colon tumor growth.

## Discussion

Although SMYD2 has been shown to play critical roles in tumor development in several types of cancers [6, 32, 34], the functional role of SMYD2 in colorectal cancer is largely unknown. Here, we identified SMYD2 as a key molecule in promoting colon tumor growth. Interestingly, we discovered a yet unknown role of SMYD2 in restricting TNF-induced apoptosis in vivo and in vitro.

Our results confirm previous reports which showed that SMYD2 is highly expressed in CRC [9]. High expression of SMYD2 has been shown to be associated with poor prognosis in several cancers, including CRC [9]. SMYD2 was identified as a methyltransferase that methylates several key cancer-involved proteins, including retinoblastoma, p53, MAPKAPK3, and STAT3, to promote cancer development [5, 8, 21, 32]. SMYD2-regulated cell proliferation is amongst these mechanisms that was reported in various types of cancers, for instance, colon cancer [9, 35]. However, a recent study using small molecule inhibitors and CRISPR/Cas9 technology targeting SMYD2 in hundreds cancer cell lines questioned the functional role of SMYD2 in tumor cell proliferation [36]. Indeed, our data support the findings and indicate that SMYD2 is dispensable for cell proliferation in colon tumor cells, as we detected similar levels of proliferation. Specifically, similar levels of ß catenin and Cyclin D1 as well as of EdU and Ki67 positive cells were detected in SMYD2 sufficient and deficient cells.

TNF is an important cytokine with direct cytotoxic activity on cells. The cytotoxic effects of TNF can be explained by its capacity to activate multiple programmed cell death pathways, such as apoptosis, necroptosis, and pyroptosis [37, 38]. It has been shown that, in mouse renal epithelial cells, TNF stimulation-induced SMYD2 production and *Smyd2* deficiency increased the levels of cell death [31]. However, the functional role of SMYD2 in regulating cell death pathways is poorly understood. The present study demonstrates for the first time that SMYD2 specifically regulates TNF-induced apoptosis and necroptosis in colon tumor cells. SMYD2 is known as a lysine methyltransferase that catalyzes the lysine methylation of histone and non-histone proteins [4–6]. Interestingly, we detected an interaction between SMYD2 and cleaved-RIPK1 in murine and human cells, suggesting that SMYD2 may directly target RIPK1. We further found that SMYD2 deficiency increased phosphorylation levels of RIPK1 under apoptotic or necroptotic stimuli. Given the crucial role of RIPK1 in the cell death pathways downstream of the TNFR, our data indicated that SMYD2 inhibits apoptosis and necroptosis, potentially by targeting RIPK1.

The induction of apoptosis and necroptosis has been considered a promising approach in cancer treatment [39]. Along the same line, SMYD2 deficiency in colon tumors showed increased cell death and restricted tumor growth. We confirmed these findings using human and murine cells in orthotopic and non-orthotopic tumor models. Since accumulating evidence suggests that TNF also has the potential of promoting tumor development under certain conditions [40], strategies to induce apoptosis or necroptosis using various downstream inhibitors of TNF without changing proliferation are being tested. We observed that, in vivo, intratumoral administration of AZ505 in the absence of TNF supplementation also induced apoptotic cell death and reduced tumor size. Furthermore, another commercially available SMYD2 inhibitor, BAY-598, also showed similar effects, suggesting that SMYD2 inhibitors may become promising candidates for colon cancer treatment. Our findings, therefore, raise the interesting possibility that SMYD2 inhibition might have therapeutic potential by inducing apoptosis and necroptosis in CRC.

In summary, our study uncovered a novel role of SMYD2 in supporting colon tumor growth. SMYD2 in colon tumor cells inhibits TNF-induced apoptosis and necroptosis. These findings suggest that targeting SMYD2 may be a potential therapeutic strategy for the treatment of colon cancer.

## Materials and methods

### Mice

Apc^Min/+^ mice, B6 /J mice and Rag1^−/−^ mice were obtained from the animal facilities of the department of medicine 1, Friedrich-Alexander-Universität Erlangen-Nürnberg (FAU). All mice were maintained under specific pathogen-free conditions and had ad libitum access to normal chow diet and sterile water. Mice of both genders aged 7−15 weeks, were randomly allocated to groups (treatment and control or recipient of control and knockout cells) in the various experiments. For ApcMin/+ mice, tumors were scored (3−5) in a blinded manner from mice randomly allocated to experimental and control groups. Experiments were performed under protocols approved by the Institutional Animal Care and Use Committee of the FAU.

### Generation of SMYD2 knockout cells

SMYD2 CRISPR/Cas9 KO Plasmid (m) (sc-432661, Santa Cruz) or SMYD2 CRISPR/Cas9 KO Plasmid (h) (sc-403129, Santa Cruz) was transfected into MC-38 cells or HT-29 cells. The HT-29 cells were purchased from ATCC and MC-38 cells were obtained from Lonza. GFP-positive cells were sorted into 96-well plates (single cell/well) by flow cytometry (FACSAria II; BD). SMYD2 knockout cell lines were established from single-cell colonies. SMYD2 deficiency was verified by western blot (WB) analysis.

### Tumor models and treatment

In the xenograft model, 2 × 10^6^ HT-29 cells were implanted into the flanks of *Rag1*^−/−^ mice via subcutaneous injection (*n* = 8/group). MC-38 cells were injected subcutaneously (1 × 10^6^ cells) into the flanks of B6/J mice (*n* = 8/group) or into the colon mucosa (1 × 10^4^ cells) of C57BL/6J mice (*n* = 5/group). Tumor size was measured at serial time points using a digital caliper and the tumor volume was calculated using *V* = (length × width × height)/2, or scored as previously described [41, 42]. Each experiments was replicated independently in other experimental models and using other cell lines as described above.

For SMYD2 inhibition in vivo, 100 µg/tumor AZ505 (HY-15226; MedchemExpress) was injected directly into tumors every two days using colonoscopy (*n* = 5/group). Tumor size was blindly scored at day 5 and day 10. In a different experiment, 50 mg/kg BAY-598 (HY-19546; MedchemExpress) was intraperitoneally injected once daily into mice bearing HT-29 xenografts (*n* = 6/group). Tumors were measured at serial time points using a digital caliper as mentioned above.

### Human material

All human material used in this study was approved by the ethics committee of the University Hospital of Erlangen.

### Cell culture

MC-38 cells and HT-29 cells were cultured in complete Dulbecco’s modified Eagle’s medium (DMEM) (31966047, Invitrogen) supplemented with 10% heat-inactivated fetal bovine serum (F7524, Sigma-Aldrich) and 1% penicillin/streptomycin (P4333, Sigma-Aldrich). All cell cultures were free of mycoplasma. Human TNF (11343015; Immunotools) was used to stimulate HT-29 cells while murine TNF (12343016; Immunotools) was used to stimulate MC-38 cells.

For 3D culture of HT-29 cells, cells were cultured with the medium mentioned above supplemented with 3 mg/ml carboxymethyl cellulose (C5013, Sigma-Aldrich).

### Cell death assay

For Lactate dehydrogenase (LDH) assay, supernatants were collected from the cultured cell. The release of LDH in supernatant was quantified via Cytotoxicity Detection Kit (LDH) (11644793001; Sigma-Aldrich) according to the manufacturer’s protocol. Supernatants from cells cultured in medium with 0.1% Triton X-100 were used as positive control and culture medium was used as negative control. The cell death was calculated as follows:

$$\% \;{{{\mathrm{cell}}}}\;{{{\mathrm{death}}}}\left({{{{\mathrm{LDH}}}}} \right) = \frac{{\left({{{{\mathrm{LDH}}}}} \right){_{{{\mathrm{sample}}}}} - \left({{{{\mathrm{LDH}}}}} \right){_{{{\mathrm{Negative}}}}}\;{_{{{\mathrm{control}}}}}}}{{\left({{{{\mathrm{LDH}}}}} \right){_{{{\mathrm{Positive}}}}}\;{_{{{\mathrm{control}}}}} - \left({{{{\mathrm{LDH}}}}} \right){_{{{\mathrm{Negative}}}}}\;{_{{{\mathrm{control}}}}}}} \times 100$$

For flow cytometry assay, cells were labeled with propidium iodide (PI) (P1304MP; Thermo Fisher) or CellEvent™ Caspase-3/7 Green Flow Cytometry Assay Kit (C10427; Thermo Fisher) and analyzed by flow cytometry (Accuri™ flow cytometers; BD) according to the manufacturer’s protocol. Experiments were performed three times and representative data are shown.

### EdU assay

Cells were labeled with EdU Proliferation Kit (ab219801; Abcam) according to the manufacturer’s protocol. Briefly, cells were cultured in medium containing 15 µM EdU for 3 h. After fixation and permeabilization, cells were further stained with iFluor 488 azide. EdU positive cells were analyzed by flow cytometry (Accuri™ flow cytometers; BD). Experiments were performed three times and representative data are shown.

### RNA extraction and real-time quantitative PCR

Total RNA was extracted from tissues or cultured cells using peqGOLD RNA Kit (732-2868, Peqlab) according to the manufacturer’s protocol and reverse transcribed into cDNA using the SCRIPT cDNA Synthesis Kit (PCR-511L, Jena Bioscience). Quantitative real-time PCR was carried out using specific QuantiTect Primer assays (Qiagen). The mRNA expression of the reference gene hypoxanthine guanine phosphoribosyl transferase (*HPRT*) was used to normalize cDNA levels.

### RNA-seq analysis

After RNA extraction and quality control, samples were sequenced using an Illumina platform generating paired-end reads. Mapping on the reference genome and quantification were done using STAR (2.7.0d) and featurecounts (v1.6.4) respectively. Differential expression of the groups of samples was performed using deseq2 (1.24.0). Enrichment, clustering, and other analysis were performed using in-house bioinformatic tools.

### Histology and immunohistochemistry

Histopathological analyses were performed on formalin-fixed paraffin-embedded tissue after Mayer’s H&E staining. Immunohistochemistry of tissue sections was performed using the TSA system as recommended by the manufacturer (NEL704A001KT, PerkinElmer). Antibodies for immunohistochemistry were as follows: anti-human SMYD2 (HPA029023; Sigma-Aldrich), anti-murine SMYD2 (21290-1-AP; Proteintech), anti-cleaved caspase-3 (9661; Cell Signaling Technology), and anti-rabbit IgG (7074; Dianova). Nuclei were stained with Hoechst 33342 (H3570; Invitrogen). Imaging was carried out using a Leica laser-scanning confocal microscope or Hamamatsu Nanozoomer 2.0 HT (Hamamatsu).

### Immunoprecipitation (IP) assay

For immunoprecipitation of complex-I, cells were seeded in 15 cm Petri dishes and treated with 1 µg/ml FLAG-TNF (ALX-522-009-C050 for MC-38 cells and ALX-522-008-C050 for HT-29 cells; Enzo Life Sciences). Whole-cell extracts were prepared in the presence of native lysis buffer (9803; Cell Signaling Technology) supplemented with protease inhibitors (Complete Mini Protease Inhibitor Cocktail, Roche) and phosphatase inhibitors (PhosphoStop Phosphatase Inhibitor Cocktail, Roche). For the control samples, 1 µg/ml FLAG-TNF was added post-lysis. Cell lysates were centrifuged for 20 min at 14,000 rpm and supernatants were collected and incubated with Anti-FLAG® M2 Magnetic Beads (M8823; Sigma-Aldrich) overnight at 4 °C. The beads were washed five times with TBS and co-precipitated proteins were eluted using SDS–PAGE loading buffer.

For immunoprecipitation of RIPK1 and SMYD2, cell lysates were prepared as mentioned above but incubated with anti-RIPK1 antibody (3493; Cell Signaling Technology) or anti-SMYD2 antibody (ab108217; Abcam) overnight at 4 °C. The next day, Protein A Agarose Beads (9863; Cell Signaling Technology) were added into the mixture and incubated in room temperature for 30 min. The beads were washed five times with native lysis buffer and co-precipitated proteins were eluted using SDS–PAGE loading buffer.

### Western blot (WB) analysis

For immunoblot analysis of lysates under non-reducing conditions, whole-cell extracts were prepared in NP40 lysis buffer (FNN0021; Thermo Fisher) supplemented with protease inhibitors (Complete Mini Protease Inhibitor Cocktail; Roche) and phosphatase inhibitors (PhosphoStop Phosphatase Inhibitor Cocktail; Roche). Supernatant proteins from cell lysates were quantified and mixed with native sample buffer (161-0738; Bio-Rad). For immunoblot analysis of lysates under reducing conditions, tissues or cell extracts were prepared in RIPA lysis buffer (89900, Thermo Fisher) supplemented with protease inhibitors and phosphatase inhibitors. Supernatant proteins from cell lysates were mixed with LDS Sample Buffer (NP0007; Thermo Fisher) and heated under 95 °C for 5 min.

Proteins were separated by SDS-PAGE using a MiniProtean-TGX gel (4−15% polyacrylamide; BioRad) and blotted on nitrocellulose membrane (Whatman) followed by incubation with the following primary antibodies: anti-SMYD2 (sc-393827; Santa Cruz), anti-caspase-3 (9662; Cell Signaling Technology), anti-cleaved caspase-3 (9661; Cell Signaling Technology), anti-HMGB1 (ab67281; Abcam), anti-MLKL (37705; Cell Signaling Technology), anti-β catenin (8480; Cell Signaling Technology), anti-RIPK1 (3493; Cell Signaling Technology), anti-TNFR (sc-8436; Santa Cruz), anti-murine pRIPK1 (53286; Cell Signaling Technology), anti-human pRIPK1 (44590; Cell Signaling Technology), and HRP-linked β actin (ab49900; Abcam). HRP-linked anti-rabbit IgG (7074; Cell Signaling Technology), anti-Rabbit IgG (Light-Chain Specific) (45262; Cell Signaling Technology), or HRP-linked anti-mouse IgG (7076; Cell Signaling Technology) was used as a secondary antibody.

### Statistical analysis and sample sizes

Significances were determined using the two-tailed students t-test for experiments with two groups. Comparisons of experiments with more than two groups were done with ANOVA. Differences were considered significant at *p < 0.05; **p < 0.01; ***p < 0.001; N.S. = not significant (*p* ≥ 0.05). Normal distribution and equal variance were assumed for all endpoints subjected to statistical testing. For cell line-based experiments, samples sizes were chosen based on power calculation and using the standard deviations calculated from preliminary experiments [43]. For these experiments, the estimated sample sizes were exceeded in order to ensure enough power. In addition, we also replicated the same experiments in another, independent knockout cell line to increase the power and the confidence in the outcomes. For studies involving mice, power and sample sizes were estimated using empirical data from several years of experience in the laboratory for the expected standard deviations involved in xenograft and transplant-mouse models of cancer and supported by the available sample size calculators. For e.g., sample size estimations for mouse tumor models were calculated to be *n* = 4, at an alpha error of 0.05 and a beta error of 0.05, at a power of 95%, and Cohens *d*. In the actual experiments, we budgeted one extra mouse for unforeseen events and used an *n* of 5 for the actual experiment. Also for all in vivo experiments, we also replicated the findings using independent mouse strains and cell lines to increase the power and confidence in experimental outcomes.

## Supplementary information

Supplementary figure legends

Suppl. Fig. 1

Suppl. Fig. 2

Suppl. Fig. 3

Suppl. Fig. 4

Supplementary Table

aj-checklist

## Data Availability

Sequencing data have been deposited at ArrayExpress under the accession number E-MTAB-11053. Full size blots have been included in the supplemental Fig. 4.
