# Supplementary material for: SMYD2 targets RIPK1 and restricts TNF-induced apoptosis and necroptosis to support colon tumor growth
Source: Cell Death Dis. 2022 Jan 12;13(1):52. doi: 10.1038/s41419-021-04483-0 (PMC8755774; doi:10.1038/s41419-021-04483-0)
Supplement: Supplementary file 1 — Supplementary figure legends [file 41419_2021_4483_MOESM1_ESM.docx]

**Supplementary Fig. 1**

(**A**) Immunoblot analysis of SMYD2 in lysates of WT and SMYD2 CRISPR/Cas9 knockout MC-38 cells and HT-29 cells, β-Actin served as a loading control. (**B-C**) WT and *Smyd2* CRISPR/Cas9 knockout MC-38 cells were injected subcutaneously into C57BL/6J mice. (**B**) Tumor growth was monitored by calipers. (**C**) Tumor weight measured at day 17 post injection.

**Supplementary Fig. 2**

(**A**) Overlapping of up-regulated and down-regulated genes from RNA-sequencing of two independent *SMYD2* knockout clones. (**B**) Representative pictures of Propidium iodide (PI) stained WT and two different clones of *Smyd2* deficient MC-38 cells stimulated with 0.1 ng/ml TNF or vehicle (mock). (**C**) Quantification of apoptotic cells as shown in (Fig 3E). (**D**) LDH release from WT and *Smyd2* CRISPR/Cas9 knockout MC-38 cells were stimulated with 0.1 ng/ml TNF in the presence or absence of 20 µM Z-VAD. DMSO was used as control. (**E**) LDH release in WT and *SMYD2* CRISPR/Cas9 knockout HT-29 cells which were stimulated with TSZ in the presence or absence of 50 µM Nec-1, 5 µM GSK-872, 1 µM Necrosulfonamide (NSA). DMSO was used as control. (**F**) Representative images of pMLKL (red) stained HT-29 tumor tissues. (**G**) **Upper panel**: LDH release from MC-38 cells stimulated with vehicle (Mock), 0.1 ng/ml TNF, 10 ng/ml TNF + 1 nM LCL161 + 20 µM Z-VAD (TSZ) for 24 h. **Lower panel**: Immunoblot analysis of RIPK3 in lysates of HT-29 and MC-38 cells, β-Actin served as a loading control. (**H**) Graph shows quantification of PI-positive cells in 3D cultured HT-29 cells.

Experiments were performed three times and representative data are shown. Data are presented as mean +SD and student’s t-test was used for statistical calculation. **P<0.01 and ***P<0.001. N.S., not significant.

**Supplementary Fig. 3**

(**A**) LDH release from HT-29 cells in the presence or absence of 20µM AZ505 were stimulated with vehicle (Mock) or 40 ng/ml TNF + 4 nM LCL161 (TS) for 24 h. (B) Cell lysates of HT-29 cells stimulated with or without 20µM AZ505 were immunoprecipitated with antibody against RIPK1 or antibody against SMYD2, followed by immunoblot analysis.

**Supplementary Fig. 4**

Full length blots and quantification for key western blots.
