# Supplementary figures and images for: SMYD2 targets RIPK1 and restricts TNF-induced apoptosis and necroptosis to support colon tumor growth
Source: Cell Death Dis. 2022 Jan 12;13(1):52. doi: 10.1038/s41419-021-04483-0 (PMC8755774; doi:10.1038/s41419-021-04483-0)

**A**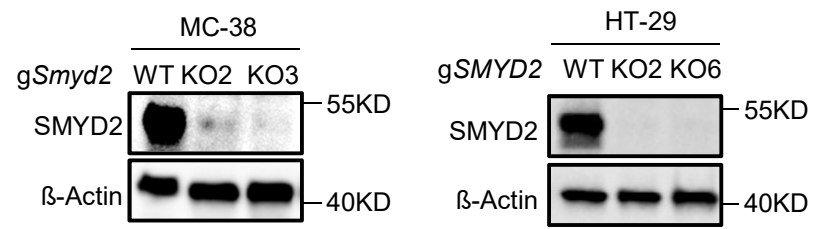**B**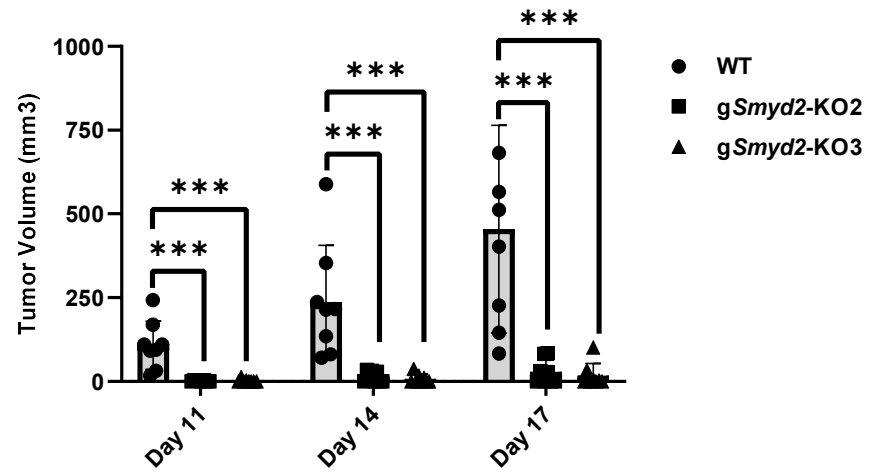**C**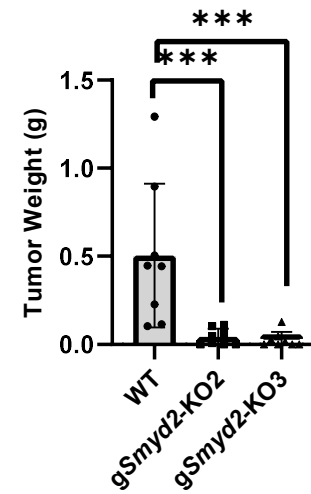

S-Fig.1

Supplement: Supplementary file 2 — Suppl. Fig. 1 [file 41419_2021_4483_MOESM2_ESM.pdf]

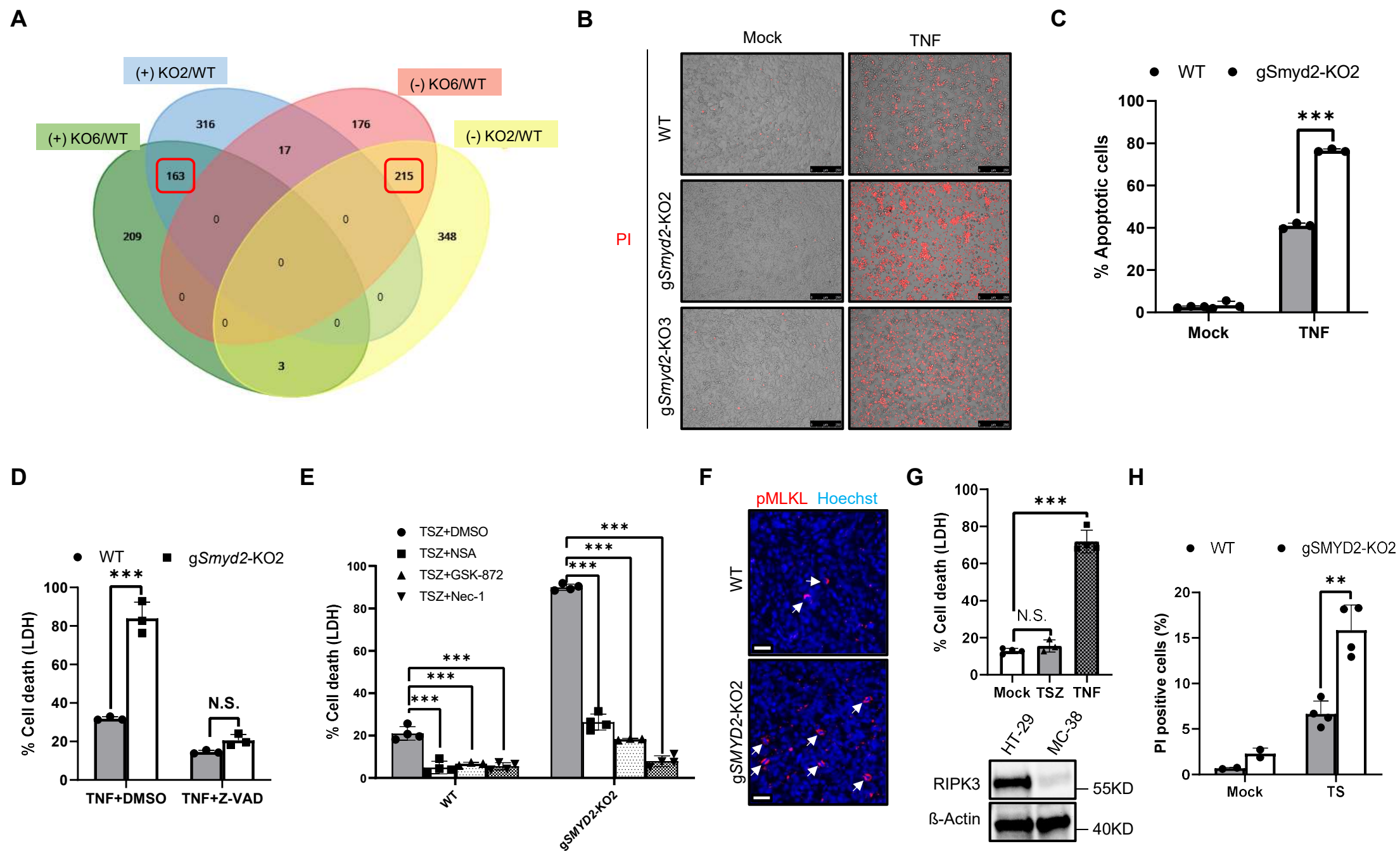

S-Fig.2

Supplement: Supplementary file 3 — Suppl. Fig. 2 [file 41419_2021_4483_MOESM3_ESM.pdf]

**A**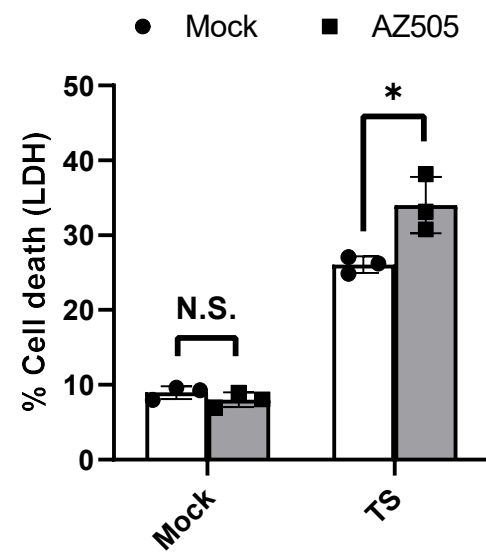**B**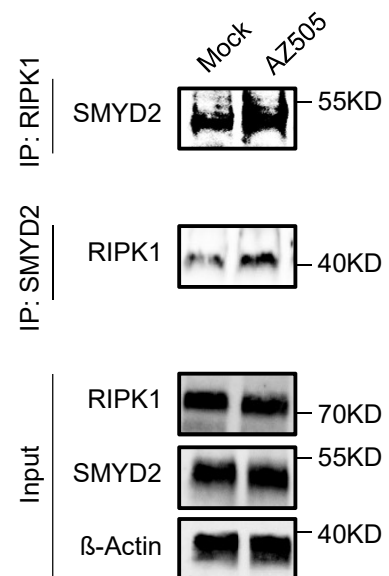

Supplement: Supplementary file 4 — Suppl. Fig. 3 [file 41419_2021_4483_MOESM4_ESM.pdf]
