# Supplementary material for: SMYD2 targets RIPK1 and restricts TNF-induced apoptosis and necroptosis to support colon tumor growth
Source: Cell Death Dis. 2022 Jan 12;13(1):52. doi: 10.1038/s41419-021-04483-0 (PMC8755774; doi:10.1038/s41419-021-04483-0)
Supplement: Supplementary file 5 — Suppl. Fig. 4 [file 41419_2021_4483_MOESM5_ESM.pdf]

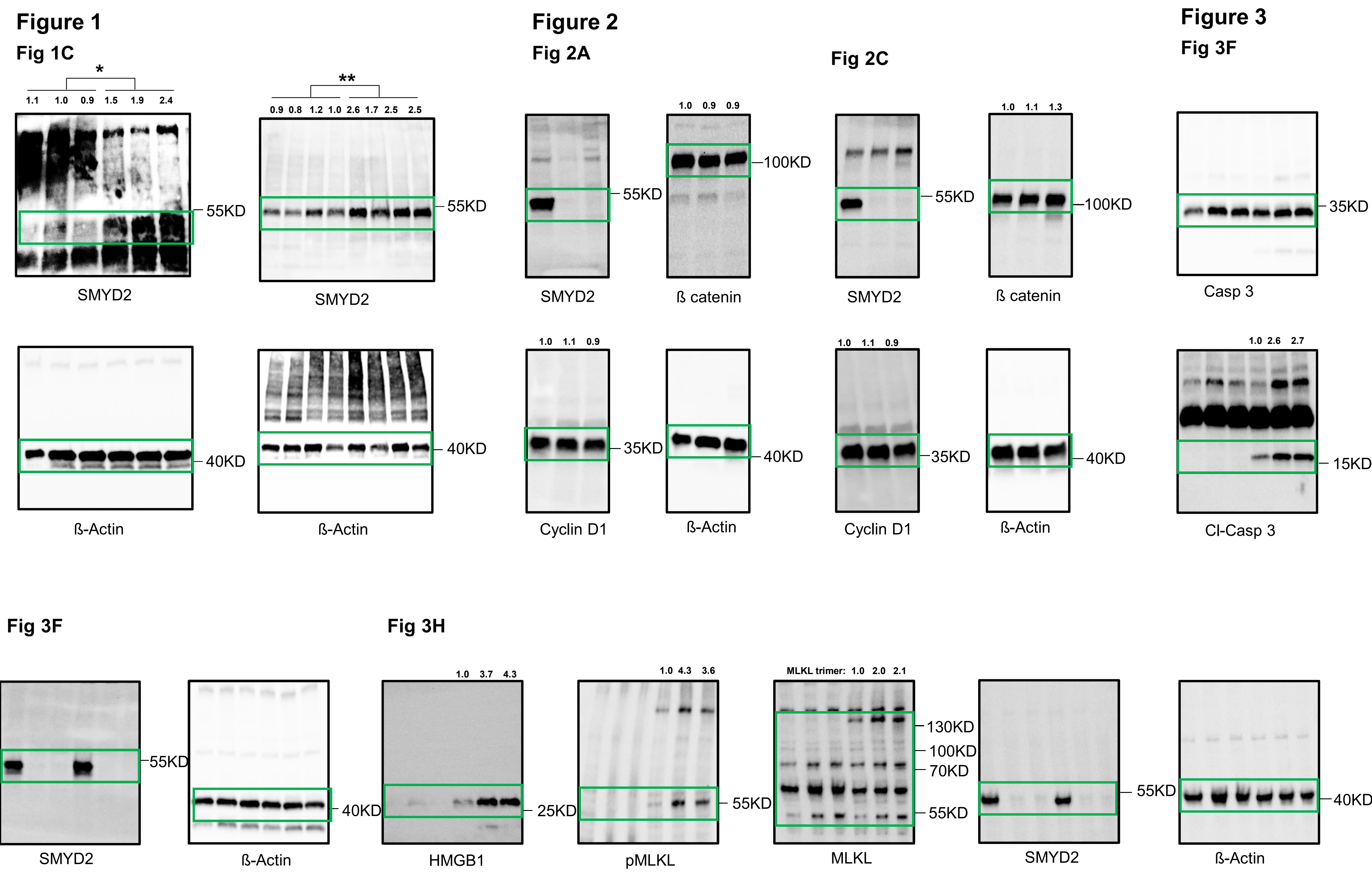

S-Fig.4: Full length blots and quantification

Figure 4

Fig 4A

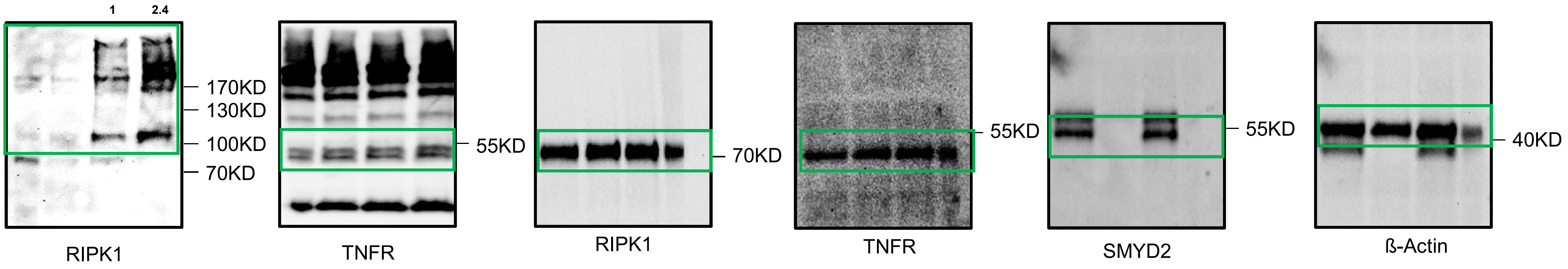

Fig 4B

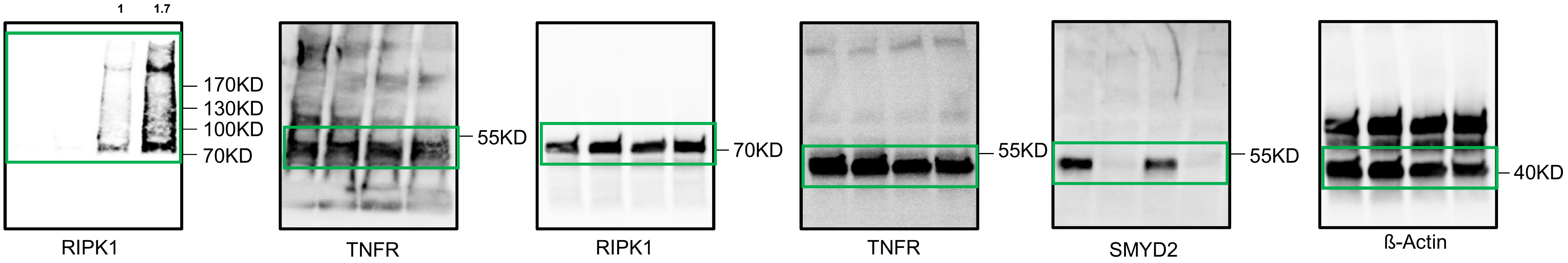

Fig 4C

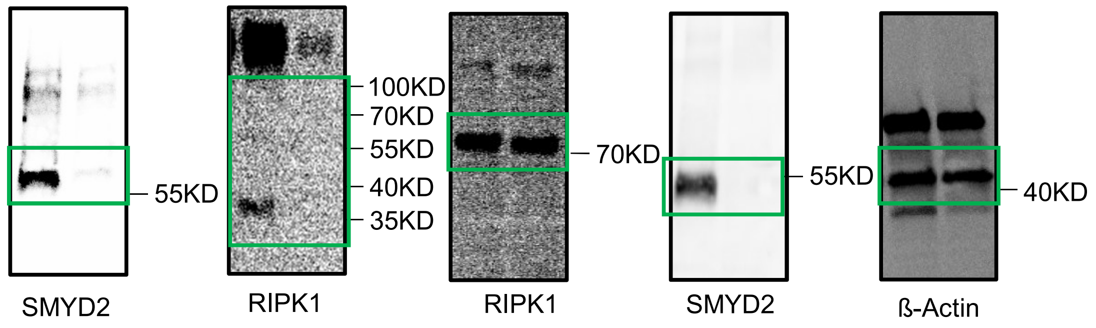

Fig 4D

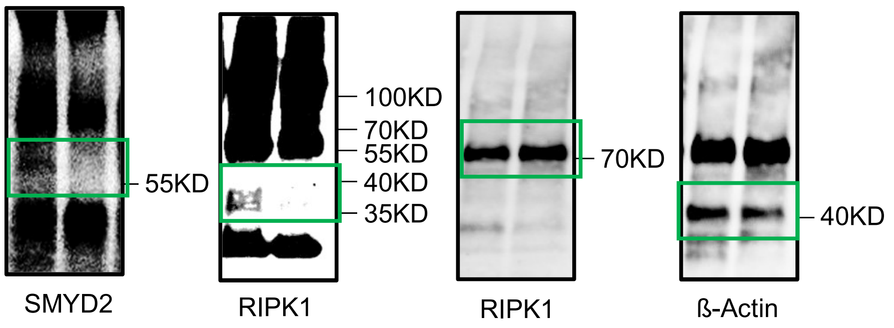

S-Fig.4: Full length blots and quantification

Figure 4

Fig 4E

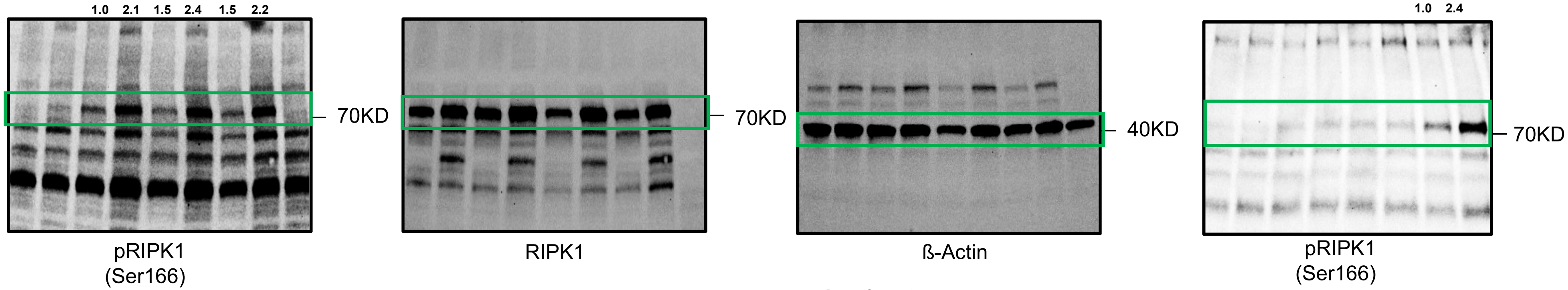

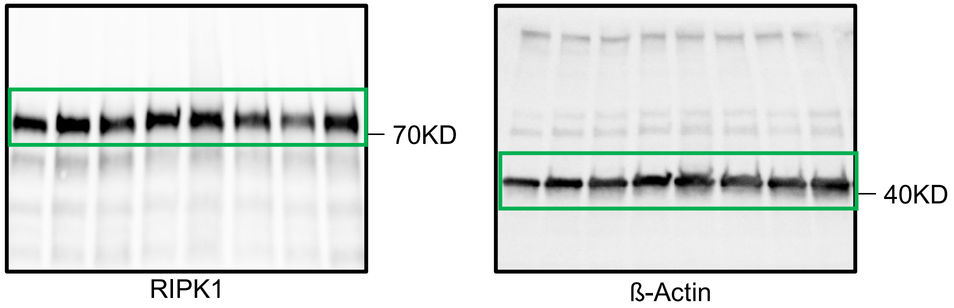

S-Fig.1A

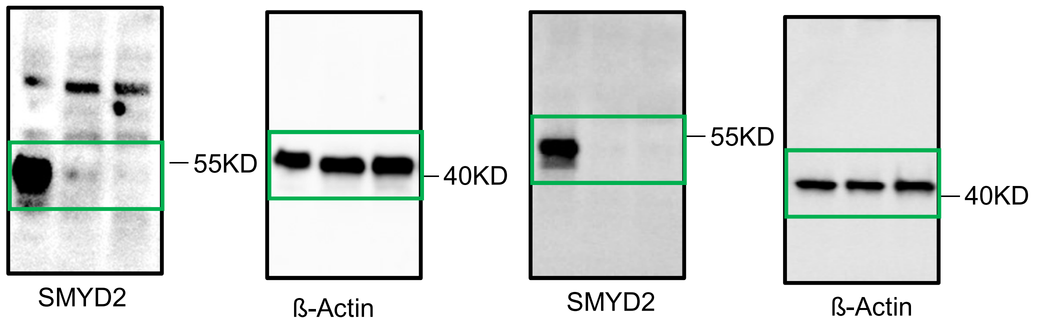

S-Fig.2G

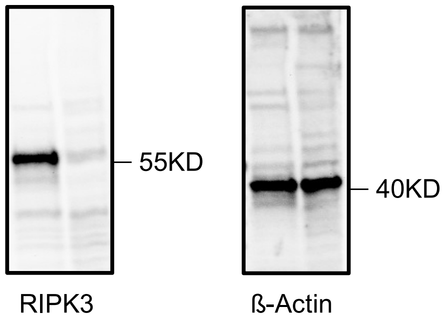

S-Fig.3A

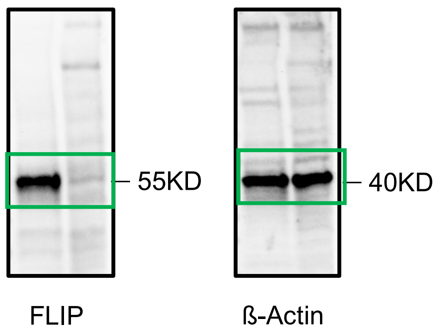

S-Fig.3C

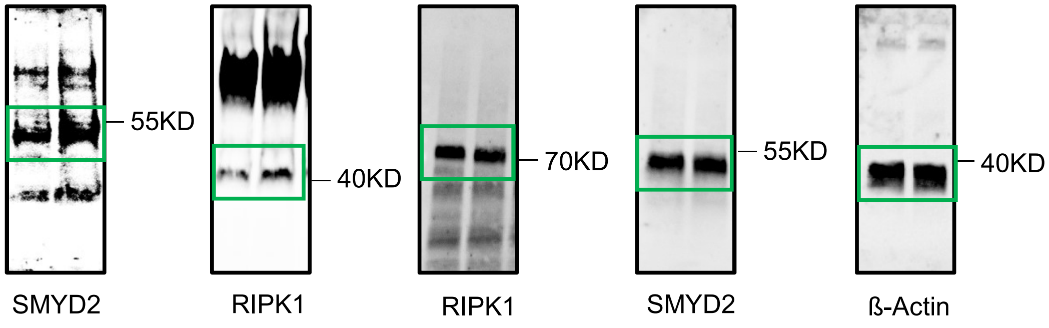

S-Fig.4: Full length blots and quantification
